# Ceramide kinase regulates acute wound healing by suppressing 5-oxo-ETE biosynthesis and signaling via its receptor OXER1

**DOI:** 10.1016/j.jlr.2022.100187

**Published:** 2022-02-24

**Authors:** Kenneth D. Maus, Daniel J. Stephenson, Anika N. Ali, Henry Patrick MacKnight, Huey-Jing Huang, Jordi Serrats, Minjung Kim, Robert F. Diegelmann, Charles E. Chalfant

**Affiliations:** 1 Department of Cell Biology, Microbiology, and Molecular Biology, University of South Florida, Tampa, FL, USA; 2 Neuroscience Drug Discovery Unit, Takeda California, San Diego, CA, USA; 3 Department of Biochemistry and Molecular Biology, Virginia Commonwealth University-School of Medicine, Richmond, VA, USA; 4 Cancer Biology and Evolution Program, The Moffitt Cancer Center, Tampa, FL, USA; 5 Research Service, James A. Haley Veterans Hospital, Tampa, FL, USA; 6 Division of Hematology & Oncology, Department of Medicine, University of Virginia, Charlottesville, VA, USA; 7 Department of Cell Biology, University of Virginia, Charlottesville, VA, USA; 8 Program in Cancer Biology, University of Virginia Cancer Center, Charlottesville, VA, USA; 9 Research Service, Hunter Holmes McGuire Veterans Administration Medical Center, Richmond, VA, USA

**Keywords:** arachidonic acid, inflammation, eicosanoids, lipidomics, group IVA phospholipases A2, 5-HETE, 5-oxo-ETE, ceramide-1-phosphate, ceramide kinase, 5-oxo-ETE, 5-Oxo-eicosatetraenoic acid, AA, arachidonic acid, C1P, ceramide-1-phosphate, CERK, ceramide kinase, CERK-KO, CERK-knockout, cPLA_2_α, cytosolic phospholipase A_2_ alpha, CPTP, C1P transport protein, COX, cyclooxygenase, EGF, epidermal growth factor, FAP, fibroblast activation protein, FLAP, 5-lipoxygenase-activating protein, HUVEC, human umbilical vein endothelial cell, LOX, lipoxygenase, NVP-231, *N*-[2-(Benzoylamino)-6-benzothiazolyl]tricyclo[3.3.1.13,7]decane-1-carboxamide, OXER1, oxoeicosanoid receptor 1, pDFs, primary dermal fibroblasts, PGE_2_, prostaglandin E_2_, PM, plasma membrane

## Abstract

The sphingolipid, ceramide-1-phosphate (C1P), has been shown to promote the inflammatory phase and inhibit the proliferation and remodeling stages of wound repair via direct interaction with group IVA cytosolic phospholipase A_2_, a regulator of eicosanoid biosynthesis that fine-tunes the behaviors of various cell types during wound healing. However, the anabolic enzyme responsible for the production of C1P that suppresses wound healing as well as bioactive eicosanoids and target receptors that drive enhanced wound remodeling have not been characterized. Herein, we determined that decreasing C1P activity via inhibitors or genetic ablation of the anabolic enzyme ceramide kinase (CERK) significantly enhanced wound healing phenotypes. Importantly, postwounding inhibition of CERK enhanced the closure rate of acute wounds, improved the quality of healing, and increased fibroblast migration via a “class switch” in the eicosanoid profile. This switch reduced pro-inflammatory prostaglandins (e.g., prostaglandin E2) and increased levels of 5-hydroxyeicosatetraenoic acid and the downstream metabolite 5-oxo-eicosatetraenoic acid (5-oxo-ETE). Moreover, dermal fibroblasts from mice with genetically ablated *CERK* showed enhanced wound healing markers, while blockage of the murine 5-oxo-ETE receptor (oxoeicosanoid receptor 1) inhibited the enhanced migration phenotype of these cell models. Together, these studies reinforce the vital roles eicosanoids play in the wound healing process and demonstrate a novel role for CERK-derived C1P as a negative regulator of 5-oxo-ETE biosynthesis and the activation of oxoeicosanoid receptor 1 in wound healing. These findings provide foundational preclinical results for the use of CERK inhibitors to shift the balance from inflammation to resolution and increase the wound healing rate.

The wound healing cascade is a dynamic process involving four distinct yet overlapping phases: hemostasis, inflammation, proliferation, and remodeling (1). Hemostasis is marked by vasoconstriction and the activation of clotting factors to reduce blood loss (2). Inflammation quickly follows to eliminate pathogens and external debris from the wound site (3, 4). Proliferation begins once foreign bodies have been removed by neutrophils and macrophages, allowing fibroblasts and keratinocytes to migrate into the wound site, ushering the transition from the inflammatory immune response and into the proliferative and angiogenetic phases (5). Lastly, new epithelial layers are formed, and collagen is cross-linked during the remodeling phase (6). Key factors in assessing wound maturation are the numbers and migration velocity of incoming fibroblasts, presence of fibroblast activation protein (FAP), and the deposition of collagen type I (7).

Our study focuses on the role of eicosanoids in wound healing, which are specialized lipid mediators with reported roles in mammalian wound response and the impairment of wound healing (8). For example, impaired wounds typically result from an imbalance between pro-inflammatory and anti-inflammatory eicosanoids such as prostaglandins and epoxyeicosatrienoic acids, respectively (9). Because of this, the blockade of cyclooxygenase-2-derived eicosanoids such as prostaglandin E_2_ (PGE_2_) is a long-used clinical technique to reduce inflammation (10). Localized excess of PGE_2_ is linked to delayed wound healing and inhibition of fibroblast function (11), while various lipoxygenase (LOX)-derived eicosanoids have been tied to increased fibroblast chemotaxis and metabolic activity (12). Furthermore, fibroblast chemotaxis during wound healing is influenced by eicosanoids through unique receptors separate from peptide-mediated chemoattraction such as platelet-derived growth factor or epidermal growth factor (13). Overall, new technological advancements in small molecule analyses (e.g., lipidomics) (14) have identified a biochemical manifestation of impaired wound healing: the development of an imbalance between pro-inflammatory and anti-inflammatory eicosanoids independent of peptide mediators (1, 2, 3, 4, 5, 6, 7, 8, 9, 10, 11, 12, 13).

The synthesis of eicosanoids begins with the initial rate-limiting step, the generation of arachidonic acid (AA) via the activity of a phospholipase A_2_ (PLA_2_) (15). One of the major PLA_2_s involved in this initial step is group IVA cytosolic PLA_2_ (cPLA_2_α), which our laboratory demonstrated is activated by direct binding to the sphingolipid, ceramide-1-phosphate (C1P) (16, 17, 18, 19, 20, 21). For example, siRNA technology to downregulate ceramide kinase (CERK), the enzyme responsible for C1P formation, blocked cPLA_2_α activation, AA release, and eicosanoid production in response to inflammatory cytokines, ATP, and calcium ionophore (16, 17). Previous findings from the Chalfant Laboratory demonstrated that the specific interaction site for C1P is localized to the calcium binding loop II of the C2 domain of cPLA_2_α, specifically the cationic β-groove (19, 20). Mutagenesis of critical amino acids for C1P interaction within this site inhibited the ability of cPLA_2_α to translocate in response to inflammatory agonists (21). These data suggest that CERK and its product, C1P, are required for the activation of cPLA_2_α, and are thus major regulators of eicosanoid synthesis in cells. Our laboratory also discovered that C1P is temporally regulated, increasing in the inflammatory phase of human wound healing (22). Additional work by our laboratory has also recently shown that the C1P:cPLA_2_α interaction negatively regulates the migration of dermal fibroblasts and 5-HETE production, and genetic ablation of this interaction enhanced acute wound healing in mice (23) (e.g. enhanced wound tensile strength, increased collagen I deposition, reduced collagen III deposition, and increased fibroblast wound infiltration).

In this study, our laboratory explored the source of C1P associated with the negative regulation of dermal fibroblast migration and wound healing. Specifically, we examined the hypothesis that inhibition of the formation of C1P via targeting the anabolic enzyme, CERK, either by genetic manipulations or by a new generation, small molecule inhibitors, will enhance the migration of murine dermal fibroblasts in culture and into the acute wounds of mice as well as induce the downregulation of PGE_2_ synthesis and upregulation of HETE production in murine dermal fibroblasts. Using a novel CERK inhibitor (SYR382141) in comparison to a conventional and established CERK inhibitor (N-[2-(Benzoylamino)-6-benzothiazolyl]tricyclo[3.3.1.13,7]decane-1-carboxamide [NVP-231]) and genetically engineered mouse models, either an ablated C1P interaction site (cPLA_2_α-KI) or *CERK* ablated (*CERK*-knockout [*CERK*-KO]), we show that inhibition/ablation of CERK confers a distinct lipid “fingerprint” consistent with dermal fibroblasts that confers more rapid cell migration and accelerates the transition from inflammation to proliferation. In expanded mechanistic studies, a distinct role for the 5-HETE metabolite, 5-oxo-eicosatetraenoic acid (5-oxo-ETE), was shown to facilitate enhanced fibroblast migration through a murine G protein-coupled oxoeicosanoid receptor (OXER1). Lastly, we found that the inhibition of CERK, postwounding, conferred enhanced wound healing and maturation providing a preclinical foundation to explore human clinical applications. Overall, these studies show that CERK-derived C1P inhibits the proliferation/remodeling stages of wound healing showing the therapeutic relevance of CERK inhibitors in this paradigm.

## Materials and methods

### SYR382141 compound

A request to access the CERK inhibitor SYR382141 should be made directly to the Neuroscience Drug Discovery Unit, Takeda California, San Diego, CA.

### PCR-based identification of WT, *cPLA*_*2*_*α*-KI, and *CERK*-KO

Genotyping of WT, *cPLA*_*2*_*α*-KI, and *CERK*-KO mice was performed by first collecting genomic DNA using an AccuStart II genotyping kit followed by PCR as previously described (24, 25) using the following primers: KI: PLA2 58534–58556, 5′-TGAGGGTCGTGCTGTAGAGTTAG-3′; PLA2 58780-58757, 5′-TGCCAGATGTGAACTTACTTCCAG-3′; KO: cre primers (5′-ATATCTCACGTACTGACGGTGGG-3′) (P1), and (5′-CCTGTTTCACTATCCAGGTTACGG-3′) (P2) (supplemental Fig. S1). Fifty nanograms of genomic DNA was used for each reaction along with the following primer concentrations, KI: 0.2 μmol/L PLA2 58534-58556 (P1), 0.2 μmol/L PLA2 58780-58757 (P2), and *CERK*-KO: 0.2 μmol/L P1 and 0.2 μmol/L P2. The following reaction cycles were repeated 33 times, 94°C for 1 min, 59°C for 1 min, and 72°C for 2 min. Reaction products expected are as follows: *cPLA*_*2*_*α*-WT: 237 bp, *cPLA*_*2*_*α*-KI: 412 bp, *CERK*-WT: 207, and *CERK*-KO: 480 bp and were examined using a 2% agarose gel.

### RT-qPCR analysis of mRNA expression

RNA from WT and *CERK*-KO primary dermal fibroblasts was converted to cDNA and used for quantitative PCR analysis using primers specific to the mouse Cerk gene (Thermo) and mouse actin control (Thermo). Methods is as previously described (26, 27, 28) (supplemental Fig. S1).

### Acute wound healing in mice

The acute wound closure rate was examined in mice as recently reported by us (23). Specifically, a 5 mm biopsy punch was performed on the dorsum of each mouse. Silicone stints were then placed around the wound, and a combination of sutures and glue was used to hold said stints in place. Wounds were dressed using Tegaderm (3M Medical) and imaged over the course of 10 days. Wound images were analyzed using the Fiji image J bundle. Wounds were tracked as percent of initial wound size over 10 days with or without CERK inhibition via small molecule inhibitor SYR382141 or genetic ablation (*CERK*-KO mouse). Treatment groups (n = 5 mice/group) are as follows: untreated, carboxymethyl cellulose sham control (1% carboxymethyl cellulose), 60 mg/kg SYR382141. The sham and SYR382141 groups received an oral gavage twice a day for nine days starting on day 1. Statistical analyses included two-way ANOVA with Tukey post hoc. A significant difference was determined by a *P* < 0.05. At the end of 10 days, tissues, blood, and wounds were harvested.

### Histology

Six millimeter samples of wound tissue were excised after 10 days; wounds were prepared for histological evaluation using the following procedure, as previously described (29). Excised wounds were fixed by placing them in 4% paraformaldehyde for 24 h; following fixation, the wound was placed in a cassette that allowed for the dehydration of the tissue, followed by clearing of the tissue using xylene (Fisher brand), and finally imbedding the tissue in paraffin wax. Sections (5 μm) of the paraffin block were placed on clear glass slides for further treatment and staining. Staining with Masson’s trichrome and hematoxylin and eosin was performed. Rabbit polyclonal anti-FAP, alpha antibody (Abcam; ab53066; 1:100) and rabbit polyclonal anti-type I collagen (Abcam ab34710; 1:200) were used in immunohistochemical staining followed by anti-rabbit secondary antibody from Vectastain Elite kit (VectorLabs PK-6100) and avidin-biotin complex enhancement. All sections were visualized with Vector NovaRED Chromogen kit (VectorLabs SK-4800) and counterstained with hematoxylin. Slides were viewed on Keyence BZ-X710 microscope and analyzed using the Fiji image J bundle for watershed cell counting or high-contrast stained area calculation, where appropriate.

### Isolation of mouse dermal fibroblast

Primary mouse dermal fibroblasts (pDFs) were isolated from 10-week-old WT, *CERK*-KO, and *cPLA*_*2*_*α*-KI BALB/c males as previously described (30). Once harvested, cells were cultured using high glucose DMEM (Gibco) supplemented with 20% FBS (Gibco) and 2% penicillin/streptomycin (Bio Whittaker) at standard incubation conditions. Cells were not used after passage 5.

### Scratch-induced mechanical trauma of fibroblasts

pDFs obtained from WT, *CERK*-KO, and *cPLA*_*2*_*α*-KI mice were plated at a density of 2 × 10^6^ on 100 mm tissue culture plates in high glucose DMEM supplemented with 10% FBS (Gibco) and 2% penicillin/streptomycin (Bio Whittaker) and left overnight to adhere at standard incubation conditions. Following the overnight incubation, cells were rested in 2% FBS (Gibco), 2% penicillin/streptomycin (Bio Whittaker), and high glucose DMEM (Gibco) for 2 h. After the 2 h resting period, mechanical trauma was induced on the monolayer by performing scratches across the diameter of the plate in an asterisk pattern using four 20 μl pipette tips on a multichannel micropipette. Media were taken for lipidomic analysis at multiple time points (0 h and 2 h).

### Exogenous addition of CERK inhibitors to human umbilical vein endothelial cells and human leukemia 60 cells

Human umbilical vein endothelial cells (HUVECs) were plated at a density of 2 × 10^5^ cells in a 6-well plate containing endothelial cell growth medium-2 with BulletKit supplements (Lonza; catalog no.: CC-3162) and allowed to rest overnight. Human leukemia (HL) 60 cells were plated at a density of 1 × 10^6^ cells in 100 mm tissue culture plates containing Iscove′s Modified Dulbecco′s Medium (ThermoFisher; catalog no.: 12200036) with 10% FBS (Gibco). After the resting period, media were aspirated and replaced with new growth media containing the addition of inhibitors (100 nM SYR382141, 300 nM NVP-231, or 0.001% DMSO control) and allowed to rest for another 24 h before media and cell lysate collection for lipid analysis, as previously described (31).

### Analysis of eicosanoids by ultra performance liquid chromatography ESI-MS/MS

Eicosanoids were separated using a Shimadzu Nexera X2 LC-30AD coupled to a SIL-30AC auto injector, coupled to a DGU-20A5R degassing unit in the following way as previously described (32). A 14 min reversed phase LC method utilizing an Ascentis Express C18 column (150 mm × 2.1 mm, 2.7 μm) was used to separate the eicosanoids at a 0.5 ml/min flow rate at 40°C as previously described by us (33, 34, 35). The column was equilibrated with 100% solvent A [acetonitrile:water:formic acid (20:80:0.02, v/v/v)] for 5 min and then 10 μl of sample was injected. Hundred percent solvent A was used for the first two minutes of elution. Solvent B [acetonitrile:isopropanol:formic acid (20:80:0.02, v/v/v)] was increased in a linear gradient to 25% solvent B at 3 min, to 30% at 6 min, to 55% at 6.1 min, to 70% at 10 min, and to 100% at 10.10 min. Hundred percent solvent B was held constant until 13.0 min, where it was decreased to 0% solvent B and 100% solvent A from 13.0 min to 13.1 min. From 13.1 min to 14.0 min, solvent A was held constant at 100%. Eicosanoids were analyzed via MS using an AB Sciex Triple Quad 5500 mass spectrometer as previously described (36). Q1 and Q3 were set to detect distinctive precursor and product ion pairs. Ions were fragmented in Q2 using N2 gas for collisionally induced dissociation. Analysis used multiple reaction monitoring in negative ion mode. Eicosanoids were monitored using precursor → product MRM pairs. The mass spectrometer parameters were as previously described (37, 38): curtain gas: 20; collisionally activated dissociation: medium; ion spray voltage: -4500 v; temperature: 300°c; gas 1: 40; gas 2: 60; declustering potential, collision energy, and cell exit potential vary per transition.

### Migration analysis of fibroblasts

Cells were seeded into 24-well tissue culture plates at a density of 7.5 × 10^4^ and allowed to grow to confluence. Once a confluent monolayer was achieved, cells were placed in 2% FBS (Gibco)/2% penicillin/streptomycin (Bio Whittaker) high glucose DMEM media and allowed to rest for 2 h. After the 2 h resting period, mechanical trauma was induced on the monolayer by performing a single scratch across the diameter of each well using a 20 μl pipette tip. Cells were observed using a live cell incubation chamber maintained at 37°C in a 95% air/5% CO_2_ atmosphere mounted on a Keyence BZ-X710 microscope, which took images every 3 min for 24 h. Migration velocity was calculated using the Keyence VW-9000 motion analysis software as previously described (23).

### Exogenous addition of eicosanoids/inhibitors on dermal fibroblasts

pDFs were seeded into 24-well tissue culture plates at a density of 7.5 × 10^4^ and allowed to grow to confluence. Once a confluent monolayer was achieved, cells were placed in 2% FBS (Gibco) high glucose DMEM media (Gibco) containing the addition of various eicosanoids and/or inhibitors at the following concentrations (1.0 nM 5-HETE, 1.0 nM 5-oxo-ETE, 7.5 nM MK886, 10 μM Gue1654, 100 nM SYR382141, 100 nM NVP-231) and allowed to rest. After the 2 h resting period, mechanical trauma was applied as mentioned previously, and the media were exchanged with fresh 2% FBS (Gibco) and high glucose DMEM media (Gibco) containing the addition of various eicosanoids at the aforementioned concentrations.

### Statistical analysis

Graphing and statistics were performed using Prism GraphPad (Prism Software, San Diego, CA). Data were analyzed via ANOVA followed by Tukey’s post hoc test or Dunnett’s multiple comparisons test where applicable. All data were reported as mean ± standard deviation (SD); *P* < 0.05 was considered statistically significant.

### Ethical considerations

All mouse studies were undertaken under the supervision and approval of the USF IACUC (Protocol# IS00004094 and IS00004110) following standards set by the Federal and State government. USF is fully accredited by AAALAC International as program #000434.

## Results

### SYR382141 inhibits C1P production in multiple cell types

**Fig. 1:**
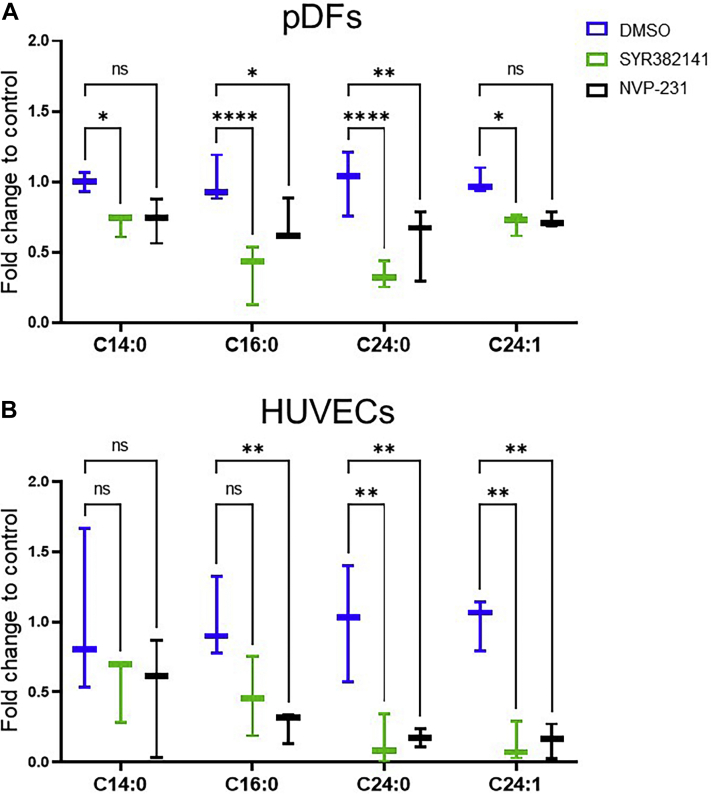
SYR382141 decreases ceramide-1-phosphate levels in cells. A: WT pDFs pretreated with SYR382141 (100 nM), NVP-231 (100 nM), or DMSO (0.01%) for 30 min received mechanical trauma via asterisk pattern scratch across the plate. Cells were collected 2 h post-injury and analyzed for C1P levels via UPLC ESI-MS/MS. Values expressed as fold change to DMSO controls (∗*P* < 0.05, ∗∗*P* < 0.01, ∗∗∗∗*P* < 0.0001; n = 3, pDFs collected from three different mice; two-way ANOVA with Dunnett's multiple comparisons test). B: HUVECs were treated with SYR382141 (100 nM), NVP-231 (300 nM), or DMSO (0.01%) for 24 h. Cells were collected and analyzed for C1P levels via UPLC ESI-MS/MS. Values expressed as fold change to DMSO controls. (∗*P* < 0.05, ∗∗*P* < 0.01, ∗∗∗*P* < 0.001; n = 3; one-way ANOVA with Tukey post-hoc test).

Previously, our laboratory reported that the interaction of C1P and group IVA PLA_2_ was a negative regulator of acute wound healing and the migration of dermal fibroblasts, but the source of C1P was not known. To examine the source of C1P for these phenotypes, we obtained a new generation inhibitor of one known source of mammalian C1P, CERK, which was developed by Takeda Corporation and designated SYR382141 and evaluated via previously described analyses (39, 40, 41, 42, 43, 44, 45). This compound inhibited CERK activity in vitro with an IC_50_ of 5 nM for human CERK and 9 nM for mouse CERK. SYR382141 did not significantly affect the activity of closely related kinases such as sphingosine kinase 1 and 2 (>100 μM, supplemental Table S1) as well as kinases in a global kinase panel (1 μM; supplemental Table S1). SYR382141 also demonstrated an IC_50_ of >30 μM to induce cytotoxicity in tissue culture (supplemental Table S1). To evaluate the ability of SYR382141 to inhibit CERK-derived C1P production in cells, pDFs) and HUVECs were treated with SYR382141 or the positive control, the CERK inhibitor NVP-231, and analyzed via ultra performance liquid chromatography coupled with electrospray ionization tandem mass spectrometry (22, 36) (Fig. 1). The levels of detectable C1P species, D-e-C_16:0_ C1P (C16:0), D-e-C_14:0_ C1P (C14:0), D-e-C_24:0_ C1P (C24:0), and D-e-C_24:1_ C1P (C24:1) were significantly reduced by SYR382141 in pDFs to an equivalent or greater extent as NVP-231 (17) using nanomolar concentrations (Fig. 1A). Similar results were observed in HUVECs (Fig. 1B). Pharmacokinetically, SYR382141 treatment of mice demonstrated significant plasma concentrations over 4 h, which would allow for initial preclinical studies on the effectiveness of inhibiting CERKin modulation of the in vivo phenotype, acute wound healing (supplemental Table S2). These data demonstrate that nanomolar concentrations of SYR382141 significantly block the production of CERK-derived C1P in cells analogous to an established inhibitor of the enzyme, but importantly, SYR382141 can be utilized to inhibit CERK in mice.

### CERK inhibition and genetic ablation improves wound closure rate and healing quality in vivo

**Fig. 2:**
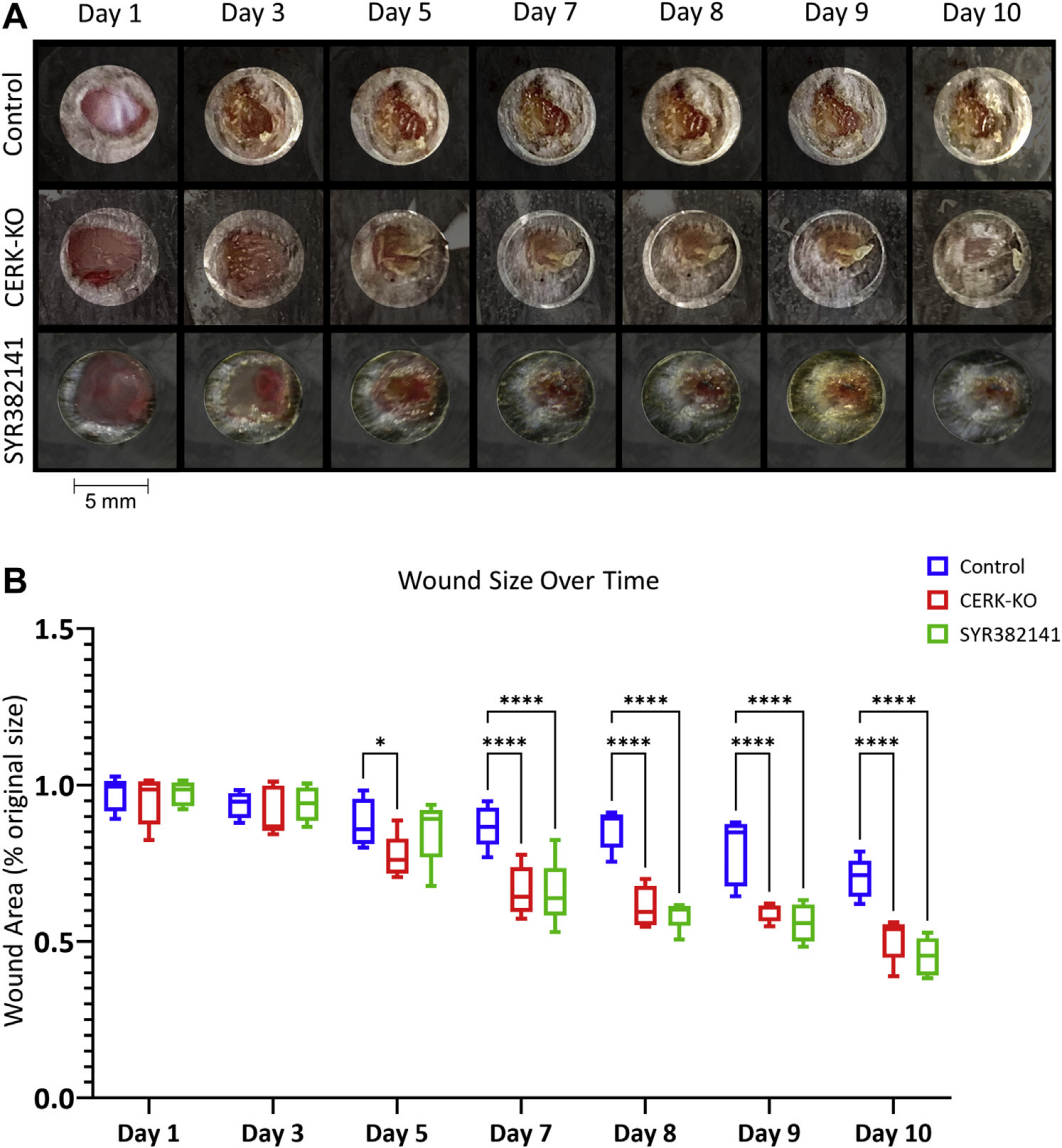
Inhibition of ceramide kinase increases the closure rate of acute wounds in mice. A: Wound closure rate of 5 mm biopsy wound on dorsum of *CERK*-KO or WT mice treated with carboxymethyl cellulose (CMC) control (1% CMC) or SYR382141 (60 mg/kg), orally twice daily beginning 1 day post-injury (1 wound per mouse repeated on two separate occasions). B: Graph depicting acute wound closure rate quantified as percent of initial wound size over 10 days. Two-way ANOVA with Dunnett's multiple comparisons test, ∗*P* < 0.05, ∗∗∗∗*P* < 0.0001; n = 5 wounds per genotype, 1 per mouse.

**Fig. 3:**
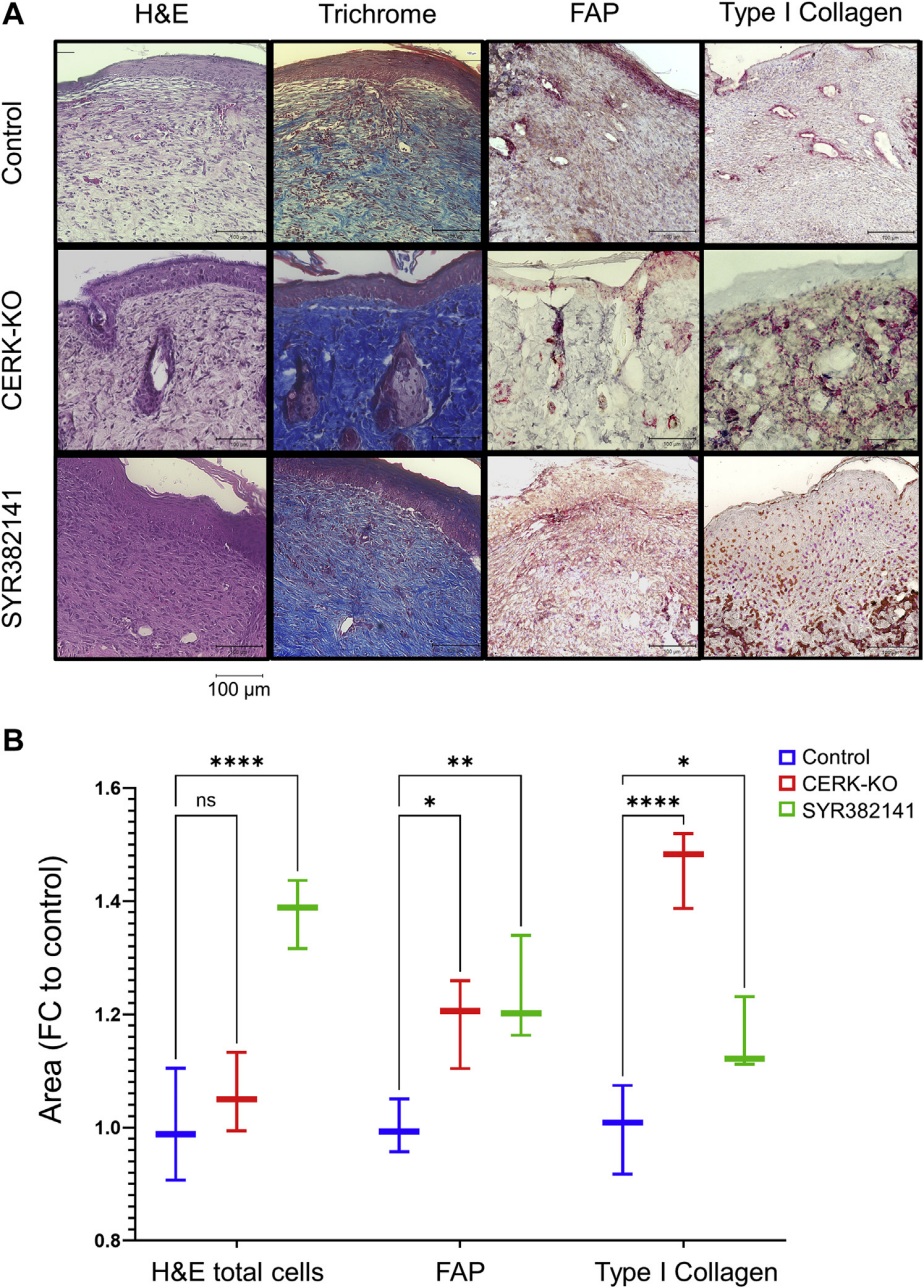
Inhibition and genetic ablation of ceramide kinase improve wound quality. A: Wound tissue harvested 10 days post-injury from WT control (1% CMC), *CERK*-KO, and SYR382141-treated (60 mg/kg) WT mice under H&E (cell infiltration), Masson’s Trichrome (collagen deposition), FAP (fibroblast activation protein), and type I collagen staining. B: Graph depicting quantification of infiltrating cells, FAP area, and type I collagen area, analyzed via ImageJ cell counter and Fiji ImageJ bundle area tool, contrast enhanced (∗*P* < 0.05, ∗∗*P* < 0.01, ∗∗∗*P* < 0.001, ∗∗∗∗*P* < 0.0001; n = 3 samples per treatment group, 1 wound per mouse; two-way ANOVA with Dunnett’s multiple comparisons test).

To determine whether CERK inhibitors could recapitulate the enhanced wound healing observed in a genetically engineered mouse model where the C1P binding site in cPLA_2_α was ablated (cPLA_2_α KI mice; KI) (23), WT mice were subjected to an acute excisional wound. One day post-wounding, WT mice were treated twice daily, orally with the new generation CERK inhibitor, SYR382141, versus the control (sham) and untreated mice. The dose of SYR382141 utilized showed significant levels of the drug in mouse tissues (e.g., kidney) (supplemental Table S3), and importantly, a significant increase in the rate of wound closure was observed after 10 days (Fig. 2). CERK inhibition dramatically increased the presence of FAP and subsequent pDFs in the acute wounds at 10 days (Fig. 3). Furthermore, both the Masson’s trichrome stain and immunohistochemistry analysis for type I collagen staining indicate enhanced collagen type 1 deposition. To confirm the specificity of the effect of SYR382141 via CERK inhibition, a novel *CERK*-KO mouse was examined in the same context, but in the absence of SYR382141 treatment (supplemental Fig. S1), which also showed a significant increase in the rate of wound closure at days 6–10 (Fig. 2) as well as enhanced pDFs (FAP staining) and collagen type 1 in the wounds (Fig. 3). These data indicate that C1P derived from CERK acts as a negative regulator of fibroblast migration into the wound environment, and inhibition or genetic ablation of *CERK* significantly enhances acute wound healing and maturation. Furthermore, inhibition of CERK is beneficial to acute wound healing in a post-wounding manner.

### CERK inhibition enhances dermal fibroblast migration

**Fig. 4:**
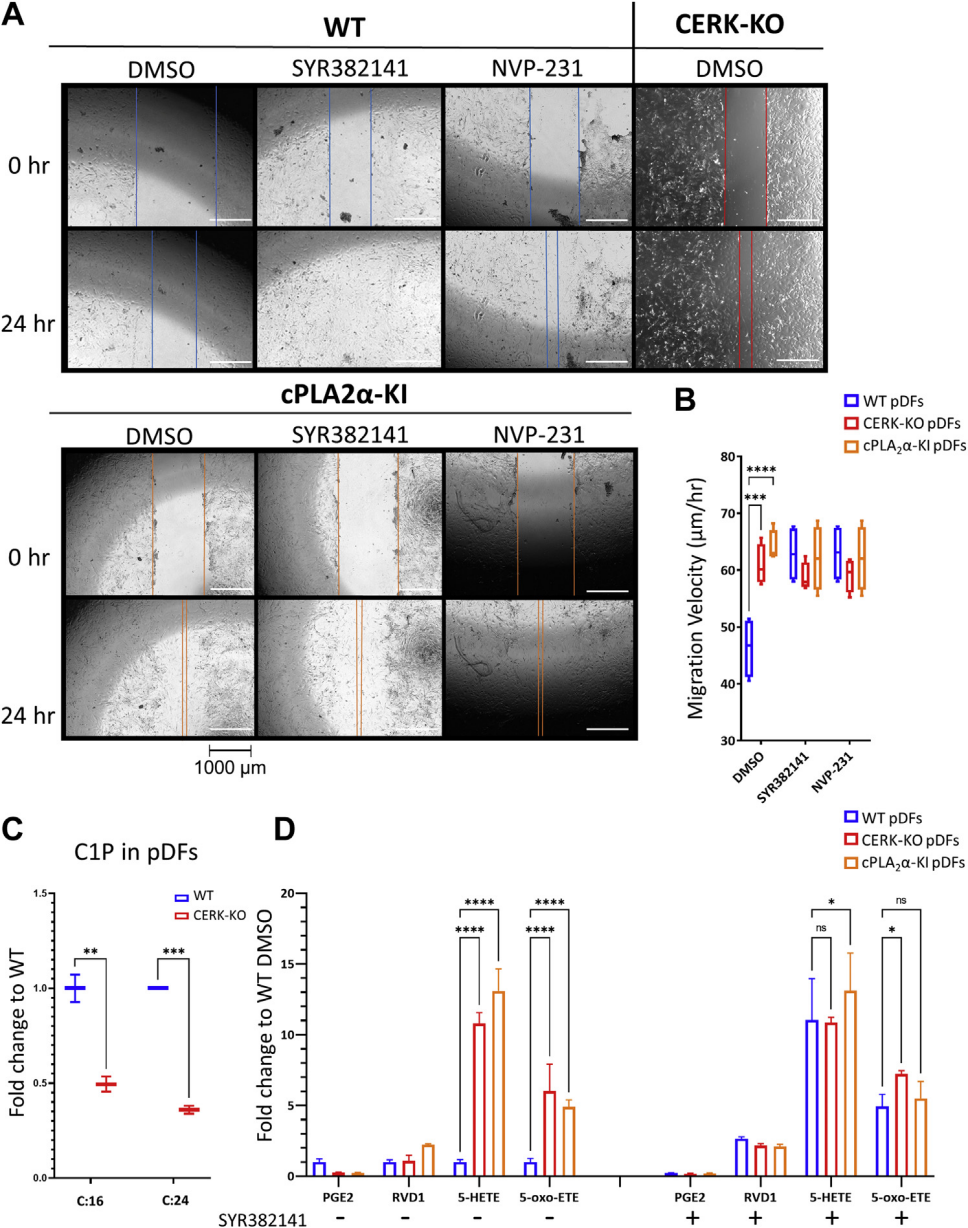
Inhibition or genetic ablation of ceramide kinase enhances the migration of dermal fibroblasts and HETE biosynthesis. A: pDFs from WT, *CERK*-KO, and *cPLA*_*2*_*α*-KI mice treated with DMSO (0.001%), SYR382141 (100 nM) or NVP-231 (100 nM). Still images from time points 0- and 24-h. Brightness enhanced; lines added for emphasis. Cells were observed using a live cell incubation chamber mounted on a Keyence BZ-X710 microscope which took images every 3 min for 24 h (n = 4; pDFs taken from two separate animals per genotype). B: Graph depicting migration velocities of pDFs treated with CERK inhibitors SYR382141 (100 nM) or NVP-231 (100 nM), calculated using the Keyence VW-9000 motion analysis software (Dunnett's multiple comparisons test; n = 4; pDFs taken from two separate animals per genotype). C: C1P (C:16 (C16:0) and C:24 (C24:0)) production in wound tissue from *CERK*-KO mice compared to WT (n = 4 per genotype; one wound per mouse). D: Eicosanoids from WT, *CERK*-KO, and *cPLA*_*2*_*α*-KI pDFs pretreated with SYR382141 or DMSO control collected 2 h after mechanical injury (two-way ANOVA with Dunnett’s multiple comparisons test; ∗*P* < 0.05, ∗∗*P* < 0.01, ∗∗∗*P* < 0.001, ∗∗∗*P* < 0.001, ∗∗∗∗*P* < 0.0001; n = 3, pDFs taken from three separate mice per genotype).

**Fig. 5:**
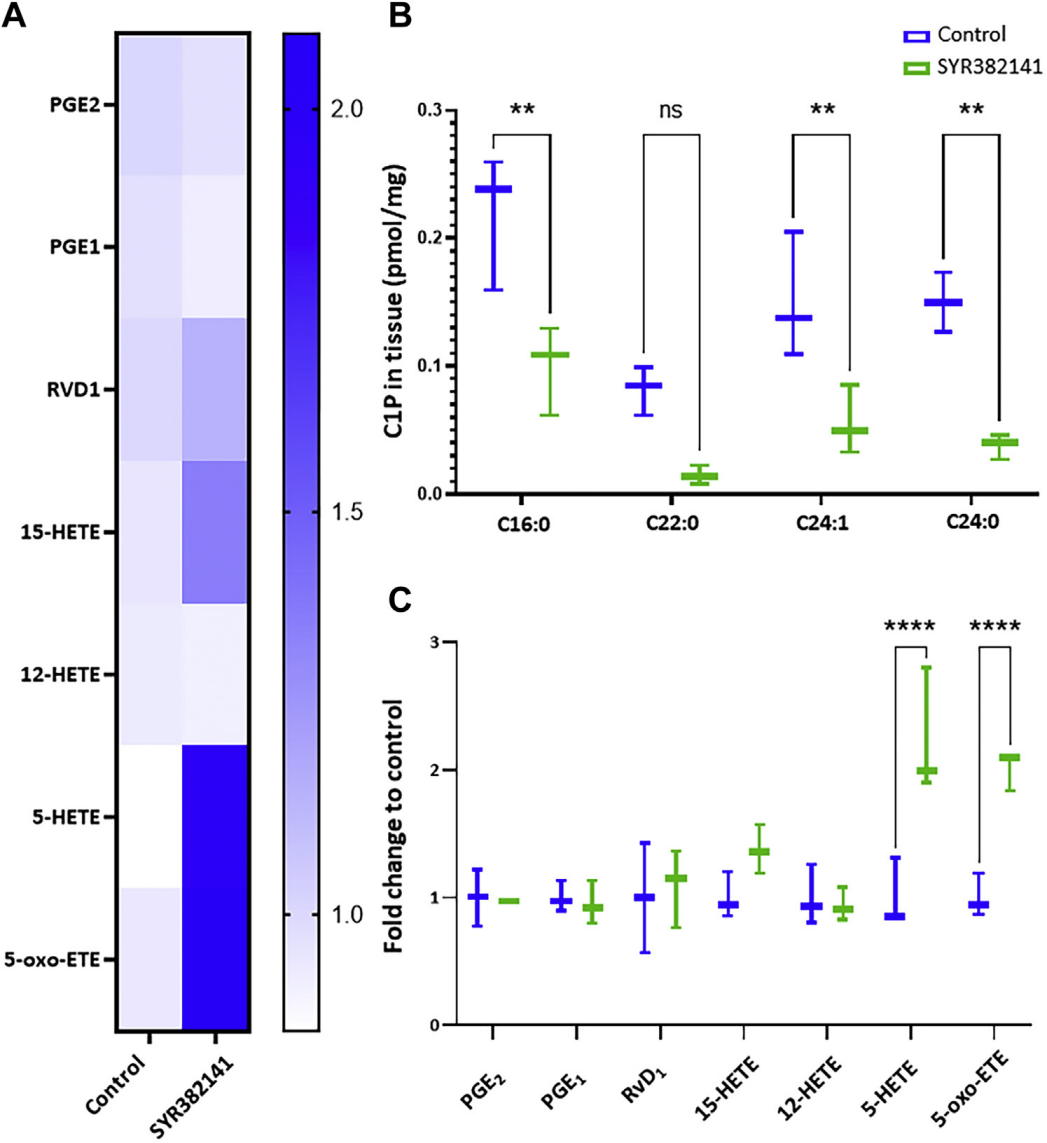
Inhibition of ceramide kinase enhances 5-HETE and 5-oxo-ETE biosynthesis in acute wounds. A: Heatmap representation of eicosanoid profile of wound tissue harvested 10 days post-injury from WT mice treated with SYR382141 (60 mg/kg) or control (1% CMC) (n = 3 per treatment group; 1 wound sample per mouse). B: Graphical comparison of C1P profile of wound tissue harvested 10 days post-injury (n = 3 per treatment; 1 wound sample per mouse; two-way ANOVA with Šídák's multiple comparisons test). C: Graph depicting eicosanoid profile of wound tissue harvested 10 days post-injury. (∗*P* < 0.05, ∗∗*P* < 0.01, ∗∗∗*P* < 0.001, ∗∗∗∗*P* < 0.0001; n = 3 per treatment group; 1 wound sample per mouse; two-way ANOVA with Šídák's multiple comparisons test).

To examine specific mechanisms that may contribute to an improved wound healing rate, pDFs were cultured ex vivo from WT and homozygous *CERK*-KO (*CERK*^−/−^; *CERK*-KO) mice along with a positive control, pDFs cultured from homozygous *cPLA*_*2*_*α* knockin mice with the C1P interaction site ablated (*cPLA*_*2*_*α* KI) (23). As previously reported (23), *cPLA*_*2*_*α*-KI fibroblasts migrated more rapidly than WT (Fig. 4A, B). The same increase in migration velocity was also observed for WT pDFs with inhibition of CERK using SYR382141 or the CERK inhibitor, NVP-231, versus sham controls (Fig. 4A, B). Additionally, pDFs from mice with *CERK* genetically ablated (*CERK*-KO), which produce a similar reduction in C1P to SYR382141-treated pDFs (Fig. 4C), also showed a similar migration velocity profile to that of SYR382141-treated WT and cPLA_2_α-KI fibroblasts (Fig. 4A, B). Also of note, addition of SYR382141 or NVP-231 was unable to further enhance the migration velocities of *cPLA*_*2*_*α*-KI or *CERK*-KO pDFs (Fig. 4B). Lipidomic analysis of SYR382141-treated WT pDFs as well as CERK KO pDFs after mechanical trauma showed significant increases in multiple LOX-derived HETE species (e.g., 5-HETE, 12-HETE, 15-HETE), and the more bioactive metabolite of 5-HETE, 5-oxo-ETE, and the pro-inflammatory prostaglandin, PGE_2_, trended downward (Fig. 4D). Changes in 5-HETE and 5-oxo-ETE production became evident as early as 60 min postinjury, but no changes in the levels of the CERK substrate, ceramide, were observed (supplemental Fig. S3A, B). Similar eicosanoid profiles were observed in HUVECs and HL-60 cells treated with NVP-231 (supplemental Fig. S2). Lipidomic analysis of wound tissue from healed wounds in SYR382141-treated mice displayed significant increases in 5-HETE and 5-oxo-ETE but did not show the reduced PGE_2_ levels observed in pDFs treated with CERK inhibitors (Fig. 5). These data demonstrate that C1P derived from the anabolic enzyme, CERK, is a negative regulator of 5-oxo-ETE biosynthesis and pDF migration via direct association with cPLA_2_α.

### Enhanced dermal fibroblast migration requires 5-HETE/5-oxo-ETE signaling via an OXER1-like receptor

**Fig. 6:**
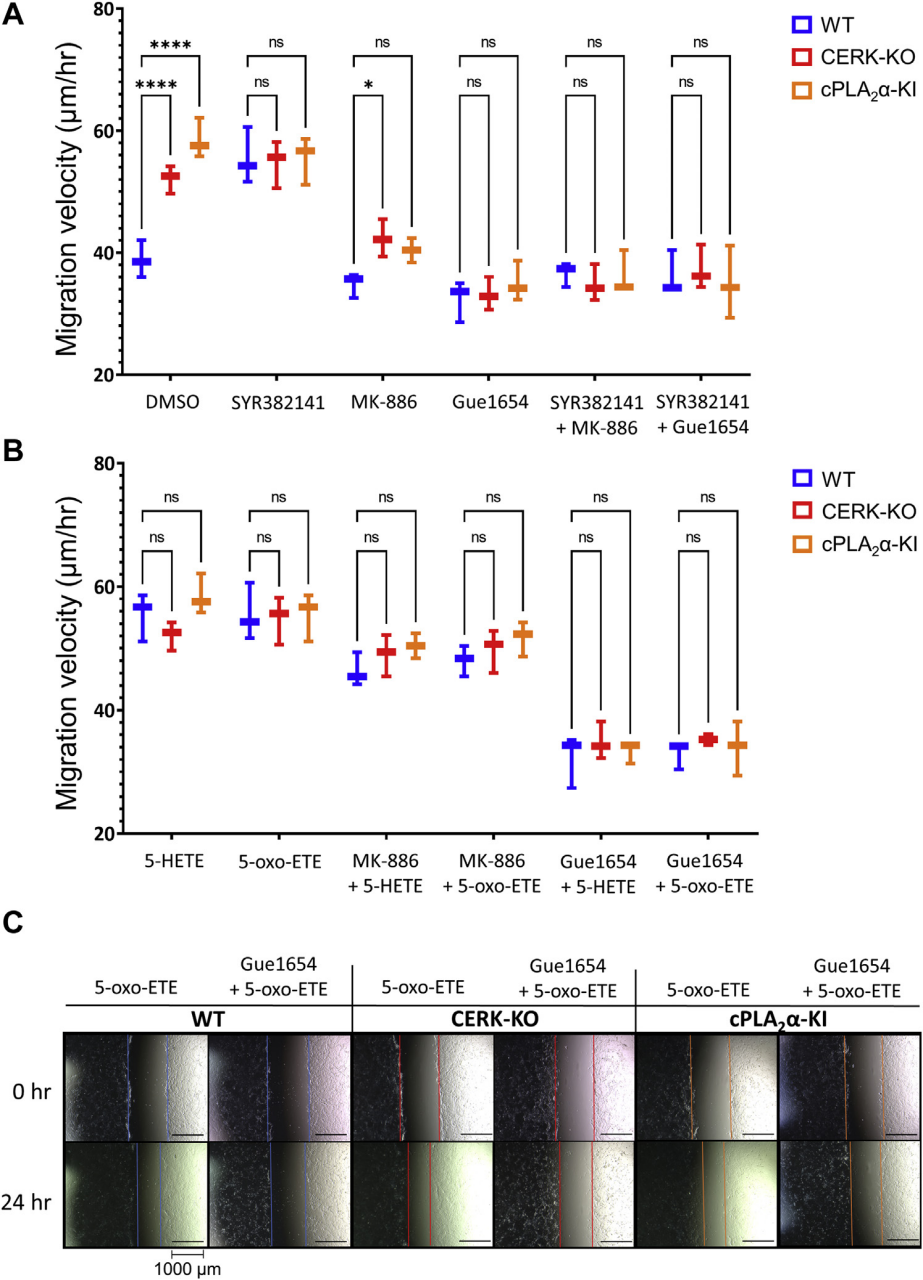
A: Graph depicting pDF migration velocity of WT, *CERK*-KO, and *cPLA*_*2*_*α*-KI pDFs treated with combinations of FLAP inhibitor MK886 (7.5 nM), OXER1 antagonist Gue1654 (10 μM), ceramide kinase inhibitor SYR382141 (100 nM). All values compared to WT DMSO control (n = 3 per treatment; pDFs taken from three separate mice per genotype; two-way ANOVA with Dunnett's multiple comparisons test; ∗*P* < 0.05, ∗∗*P* < 0.01, ∗∗∗*P* < 0.001, ∗∗∗∗*P* < 0.0001). B: Graph depicting pDF migration velocity of WT, *CERK*-KO, and *cPLA*_*2*_*α*-KI pDFs treated with combinations of 5-HETE (100 nM), and 5-oxo-ETE (1 nM) treatments in combination with MK886 (7.5 nM) and Gue1654 (10 μM). All values compared to panel (A) WT DMSO control (n = 3 per treatment; pDFs taken from three separate mice per genotype; two-way ANOVA with Dunnett's multiple comparisons test; ∗*P* < 0.05, ∗∗*P* < 0.01, ∗∗∗*P* < 0.001, ∗∗∗∗*P* < 0.0001). C: Representative microscope images of 5-oxo-ETE rescue and Gue1654 suppression of pDF migration from data graphed in panels A and B. Contrast enhanced, lines added for emphasis.

To determine whether the 5-HETE/5-oxo-ETE derived via the 5-LOX/5-lipoxygenase-activating protein pathway drives pDF migration in a paracrine/autocrine manner, we employed the 5-lipoxygenase-activating protein inhibitor MK886 to prevent 5-HETE/5-oxo-ETE production and Gue1654, a non-Gα_i_-biased antagonist of human OXER1, which blocks 5-oxo-ETE-triggered functional events (46). MK886 and Gue1654 effectively reduced *cPLA*_*2*_*α* KI and *CERK*-KO pDF migration velocity to that of WT pDFs and blocked SYR382141 effects on the WT pDFs (Fig. 6). A combination of MK886 and Gue1654 did not act additively or synergistically to block the effect of the genetic ablation of the C1P/cPLA_2_α interaction or CERK inhibition. These data demonstrate that 5-HETE/5-oxo-ETE drive the enhanced migration in cPLA_2_α KI and SYR382141-treated or *CERK*-ablated pDFs via autocrine/paracrine signaling through a murine G-protein-coupled OXER1.

## Discussion

In this study, we characterized both a new *CERK*-KO mouse and a new small molecule inhibitor of CERK in the context of an enhanced wound healing phenotype. Specifically, we showed that inhibition of CERK-derived C1P could recapitulate the finding that genetic ablation of the C1P/cPLA_2_α interaction site enhances acute wound healing through increased dermal fibroblast migration and accelerated type I collagen deposition characteristic of nonfibrotic healed wounds (22). In this regard, *CERK* ablation or inhibition with this novel compound did recapitulate enhanced acute wound healing, and importantly, this effect occurred post-wounding and thus sets the foundational preclinical studies to move forward for future clinical efficacy studies. Furthermore, this study determines the source of C1P activating cPLA_2_α signaling in suppressing dermal fibroblast migration and wound maturation as CERK versus another means of C1P biosynthesis like the reported mammalian S1P acylase activity (47).

**Fig. 7:**
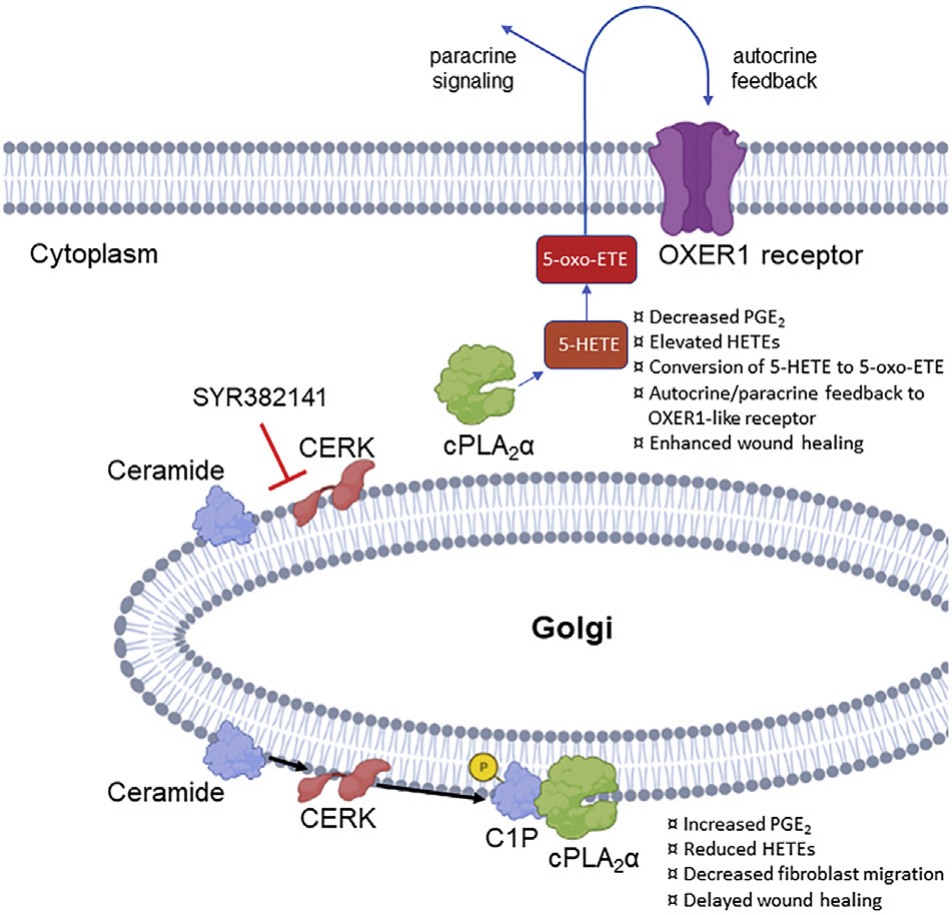
Inhibition of CERK-induced C1P elevates 5-HETE and its conversion to 5-oxo-ETE and subsequent action on OXER1-like receptor resulting in increased fibroblast activity and expedited wound healing. The presence of CERK results in elevated C1P, leading to an eicosanoid shift from HETEs to PGE_2_ resulting in delayed wound healing via reduced fibroblast migration.

This study also expanded the mechanistic knowledge as to how the association of CERK-derived C1P with cPLA_2_α drives enhanced wound healing, wound maturation, and pDF migration/collagen deposition. More specifically, this study demonstrated that CERK-derived C1P negatively regulates 5-HETE biosynthesis with 5-HETE, but also showed that 5-HETE was metabolized to 5-oxo-ETE, which is 100-fold more biologically active. This 5-HETE metabolite was found to act in an autocrine/paracrine manner by activating a murine OXER1 receptor to enhance pDF migration (Fig. 7). The murine OXER1 receptor has yet to be defined unlike the homolog to the human OXER1 receptor. Regardless, the effectiveness of the OXER1 antagonist, Gue1654, which blocks both 5-HETE and 5-oxo-ETE effects on pDF migration, shows the existence of an undefined homolog of the human OXER1. A recent report by Lai *et al.* (48) also demonstrates that the uncharacterized murine OXER1 receptor does exist in mice due to successful treatment with Gue1654 affecting coronary artery ligation-induced ischemic myocardial injury. Homology analysis shows that the mouse hydroxycarboxylic acid receptor 2 (HCAR2), which has been proposed to mediate 5-oxo-ETE responses in mice (49) and shares approximately 42% homology with human OXER1, is highly expressed in adipose tissue and macrophages and is expressed 3- to 5-fold higher when exposed to inflammatory mediators (e.g., lipopolysaccharide, tumor necrosis factor α, interleukin 1) (50). However, future studies need to confirm this murine receptor as the target for Gue1654 in mice to block 5-HETE and 5-oxo-ETE biological responses. Preliminary studies from our group utilizing compounds reported to nonspecifically downregulate murine HCAR2 in pDFs show similar migration inhibition effects as Gue1654 supporting the hypothesis that HCAR2 is the 5-oxo-ETE receptor in mice. Overall, this study can conclude that an OXER1 G-protein-coupled receptor exists in mice, which is required for 5-HETE and 5-oxo-ETE to enhance pDF migration, but this study cannot confirm the exact receptor homolog to human OXER1 at this time.

Notably, both the loss of C1P/cPLA_2_α interaction and the inhibition of CERK resulted in dramatic decrease of inflammatory prostaglandins concomitant with 5-HETE increase. Indeed, C1P reportedly increases during the inflammatory stage of wound healing in human subjects but then decreases during the proliferation and remodeling stages (51). These findings, when coupled with C1P being a negative regulator of proliferation and wound maturation (23), suggest that C1P is a pro-inflammatory mediator modulating the inflammatory stage and an essential “stop-gap” in the subsequent activation of the proliferation stage. Thus, blocking C1P production or C1P interaction with cPLA_2_α proves to be beneficial for condensing the inflammatory phase and inducing the proliferation/remodeling phases earlier by enhancing fibroblast activity. Interestingly, CERK inhibitors also increased other proresolution 15-LOX-derived eicosanoids such as 15-HETE and RVD_1_ in various cell types. These data further suggest that C1P may regulate additional PLA_2_ isoforms, which regulate the production of these specific eicosanoids. One possibility could be cPLA_2_β, which has not been well characterized and contains a C2-domain analogous to cPLA_2_α (52, 53).

One conundrum from our study is the observation that inhibition of C1P improves pDF migration. Although congruent with our previous finding in mouse embryonic fibroblasts with *CERK* ablated, other laboratories have reported that C1P treatment induces cellular migration in macrophages (54) and pancreatic cells (55). We hypothesize that these differences may be due to the cellular localization of C1P. For instance, exogenous C1P treatment using a vesicle-based delivery mainly increases the C1P content of the plasma membrane (PM) in various cell types (56) with rapid metabolism to ceramide observed (36). This PM pool of C1P may encourage migration via association with factors such as annexins (32). Additionally, the reported opposing functions of C1P on cell migration may be due to cell type–specific variances as in our studies. Indeed, our studies utilize fibroblasts, whereas other reports show that C1P enhances macrophage migration (54). Inhibition of C1P may reduce macrophage-induced inflammation in the wound, which is a plausible mechanism to accelerate dermal wound healing. Our current study argues against a cell type–specific variance as we show that both HUVECs and HL-60 cells increase 5-HETE and 5-oxo-ETE in response to CERK inhibition, and thus, any context variations are likely due to differences in OXER1 activation in specific cell types.

Metabolism to other sphingolipids that drive cellular migration may also be a plausible explanation for the differential reports on C1P in cell migration (e.g., catabolism to sphingosine-1-phosphate) (57). Furthermore, an additional anabolic pathway for C1P generation does exist in mammalian cells, which may involve an sphingosine-1-phosphate-acylase (47) at the PM. CERK-derived C1P is more recognized as a Golgi form of C1P (58) and associated with cPLA_2_α activation and eicosanoid biosynthesis. The form of C1P may inhibit migration due to association with cPLA_2_α localized to this organelle and modulation of specific eicosanoids (e.g., PGE_2_) (58). Additionally, transport of C1P to the PM by C1P transport protein (CPTP) remains a plausible regulatory mechanism between Golgi-C1P blocking migration and PM-C1P enhancing migration or by limiting C1P release for paracrine effects (59, 60). CERK regulation and C1P anabolism are still an enigma in the field, which was mainly due to a lack of sensitive techniques to consistently measure C1P levels accurately in cells. Although this issue is now rectified, C1P biosynthesis has not been strongly revisited. Originally, due to the very low levels of C1P in the cells versus the substrate of CERK, ceramide, conversion to C1P was not considered a plausible cellular “rheostat” for reducing ceramide levels to block proapoptotic mechanisms. The discovery of the CPTP in 2013 dispelled that dogma as these studies found a high level of “flux” of ceramide through CERK in some cell types, which was rapidly transported from the Golgi to other cellular organelles and catabolized (59). In this study, no increases in ceramides in the time frame (60 min) that C1P increases by ∼2-fold was observed. Inhibition of CERK caused a marked trend in the increase of ceramide levels after mechanical trauma to cells in culture, but these differences were not significant suggesting either CERK activation or CPTP inhibition/downregulation is the mechanism of C1P induction. Nonetheless, the unique functions for specific C1P anabolic pathways require further study to elucidate their specific cellular roles, which may be opposing depending on the topology of the C1P production or cell type–specific. With recent advances in examining metabolic “flux” of sphingolipids via mass spectrometry, the regulatory mechanisms for C1P biosynthesis can be explored in the future in detail.

One of the more important outcomes of this study is the demonstration of rapid acute wound closure from C1P inhibition in vivo in a postwounding manner. Thus, topical treatment of wounds with a CERK inhibitor could be effective in enhancing wound healing and possibly even incorporated into antibiotic ointments in future studies. One of the unique strengths of SYR382141 is its ability to significantly reduce C1P levels in vivo. Additionally, CERK inhibiting drugs may also be adaptable to chronic wounds, which fail to resolve and are “stalled” in the inflammatory stage possibly due to continued C1P production, high PGE_2_ (61), and elevated neutrophil activity (62). Indeed, the main component of the venom of the Brown Recluse spider (63) is sphingomyelinase D (SMase D), which hydrolyzes sphingomyelin to C1P. The dermal necrosis/ulceration induced by SMase D is well documented in patients bitten by the Brown Recluse spider (64), which presents as ulcerative wound similar in many aspects to a pressure ulcer. Thus, the chronic synthesis of C1P would be a plausible driver of “stalled” wound healing. CERK inhibitors may have the ability to suppress the synthesis of C1P as well as PGE_2_ (65), enhance 5-HETE and 5-oxo-ETE production, and induce fibroblast activation, thus promoting wound healing of stall wounds linked to neutrophilia and a chronic inflammatory stage. On the other hand, some pathogenic bacteria also have SMase D (66, 67), which may stall wound healing independent of CERK, and thus, a combination therapy of 5-oxo-ETE and CERK inhibitors may provide more benefit in a clinical setting where the wound microbiome is also a major factor in wound healing outcomes. Lastly, a better mechanistic understanding of the CERK/5-oxo-ETE interplay could be valuable for treating other diseases highly correlated to these lipid biomarkers such as preeclampsia (68, 69) and type 1 diabetes (70, 71).

In conclusion, this study demonstrates that enhanced wound healing and maturation is induced by blocking CERK-derived C1P, which is a negative regulator of fibroblast function and the 5-HETE/5-oxo-ETE/OXER1 axis. Our findings show the importance of sphingolipids and resulting eicosanoids in the wound healing process and provide the groundwork for foundational preclinical studies to move forward for future clinical therapeutic development of CERK inhibiting drugs that accelerate the healing rate and closure of wounds, especially for postinjury treatment.

## Data availability

All data are contained within the manuscript.

## Supplemental data

This article contains supplemental data (72, 73, 74, 75, 76, 77, 78, 79, 80).

## Conflict of interest

The authors declare that they have no conflicts of interest with the contents of this article.
