# Supplementary material for: Ceramide kinase regulates acute wound healing by suppressing 5-oxo-ETE biosynthesis and signaling via its receptor OXER1
Source: J Lipid Res. 2022 Feb 24;63(4):100187. doi: 10.1016/j.jlr.2022.100187 (PMC8980959; doi:10.1016/j.jlr.2022.100187)
Supplement: Supplemental Figures S1–S3 and Tables S1–S3 [file mmc1.pdf]

SUPPLEMENTAL INFORMATION:

Ceramide kinase regulates acute wound healing by suppressing 5-oxo-ETE biosynthesis and signaling via its receptor OXER1.

Kenneth D. Maus[1], Daniel J. Stephenson[1], Anika N. Ali[1], H. Patrick MacKnight[1], Huey-Jing Huang[2], Jordi Serrats[2], Minjung Kim[1], Robert F. Diegelmann[3], and Charles E. Chalfant[1,4-9]

[1]Department of Cell Biology, Microbiology, and Molecular Biology, University of South Florida, Tampa, FL 33620

[2]Neuroscience Drug Discovery Unit, Takeda California, San Diego, CA, USA (J.S.), Present address: Engrail Therapeutics, San Diego, CA, USA (JS), Present address: ADARx Pharmaceuticals, San Diego, CA 92121 (H-JH)

[3]Department of Biochemistry and Molecular Biology, Virginia Commonwealth University-School of Medicine, Richmond, VA 23298-0614

[4]Cancer Biology and Evolution Program, The Moffitt Cancer Center, Tampa, FL 33620

[5]Research Service, James A. Haley Veterans Hospital, Tampa, FL 33612

[6]Department of Medicine, Division of Hematology & Oncology, University of Virginia, Charlottesville, VA, 22903

[7]Department of Cell Biology, University of Virginia, Charlottesville, VA, 22903

[8]Program in Cancer Biology, University of Virginia Cancer Center, Charlottesville, VA, 22903

[9]Research Service, Hunter Holmes McGuire Veterans Administration Medical Center, Richmond VA, 23298

Supplemental Material

Table S1. SYR382141 compound *in vitro* and intracellular cytotoxicity studies. Recombinant human and mouse ceramide kinase were assayed in the presence and absence of various concentrations of SYR382141 as previously described by others[72-74] using the ADP-Glo™ Kinase Assay (Promega Corporation), The closely related lipid kinases, sphingosine kinase 1 and 2 (SPHK1, SPHK2), phosphatidylinositol 4-kinase beta (PIK4B), phosphatidylinositol-4-phosphate 3-kinase catalytic subunit type 2 alpha (PI3KC2A), and phosphatidylinositol 3-kinase catalytic subunit type 3 (PIK3C3), were also analyzed in the presence and absence of various concentrations of SYR382141 also using the ADP-Glo™ Kinase Assay (Promega Corporation). The effect of SYR382141 on a Global kinase panel[75] was also assessed. Lastly, cytotoxicity of SYR382141 was assessed in HepG2 cells via MTT assay as previously described[76-80]. Data are courtesy of Takeda Corporation.

Compound	SYR382141
Hm Enz IC_{50} (nM)	5
Ms Enz IC_{50} (nM)	9
SPHK1/SPHK2/PI4KB/PI3KC2A/PIK3C3 IC_{50} (μM)	>100
Global Kinase panel ≤ 1 μM	Negative
Cytotox 72h IC_{50} (μM)	30.3

Table S2. Pharmacokinetics of SYR382141 in mice. The concentration of SYR38214 was analyzed in the plasma of mice retention in mouse plasma over the indicated time using UPLC-ESI-MS/MS following oral gavage of the compound at the indicated concentration. Data are courtesy of Takeda Corporation.

**SYR382141 (15 mg/kg)**			
Time (hr)	Mean Plasma Conc. (ng/mL) Total	Mean Plasma Conc. (nM) Total	Mean Plasma Conc. (nM) Free
0.5	5114	12247	73
1	4771	11426	69
4	586	1403	8
**$f_{u,plasma}$ = 0.006**			

Table S3. Retention of SYR382141 in kidney. SYR382141 compound present in mouse kidney tissue after 10 days PO b.i.d.

	Untreated			Sham			60 mg/kg		
	SYR382141 conc.		SD	SYR382141 conc.		SD	SYR382141 conc.		SD
Kidney (ng/g)	0.000	±	0.000	0.000	±	0.000	38.780	±	12.968

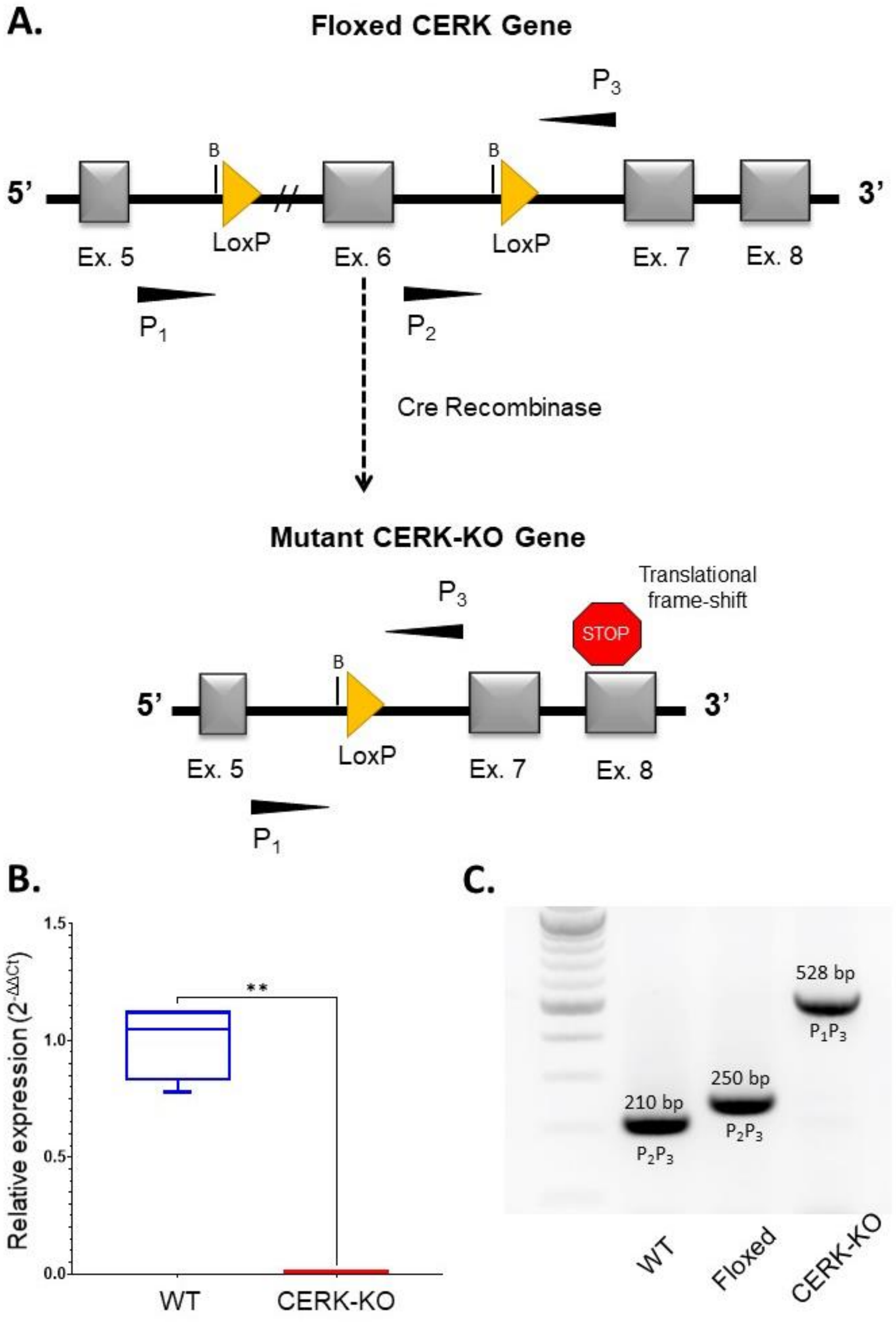
A.
Floxed CERK Gene
P3
B
B
5'
3'
LoxP
LoxP
Ex. 5
Ex. 6
Ex. 7
Ex. 8
P1
P2
Cre Recombinase
Mutant CERK-KO Gene
Translational
frame-shift
P3
STOP
B
5'
3'
LoxP
Ex. 5
Ex. 7
Ex. 8
P1
B.
Relative expression (2-ΔΔCt)
1.5
1.0
0.5
0.0
**
WT
CERK-KO
C.
528 bp
P1P3
250 bp
P2P3
210 bp
P2P3
WT
Floxed
CERK-KO

Figure S1. Strategy and design of the novel *CERK* KO mice. (A) A schematic representation of the inserted flanked loxP sites ("Floxed") and their associated BamH I sites at the beginning of endogenous introns 5 and 6 used to generate the *CERK* knockout (*CERK*-KO) mice used in this study. Mice possessing a floxed *CERK* allele were bred to mice expressing Cre recombinase in the germline (EIIa-*cre* homozygotes, JAX stock #003724). Offspring were screened for the KO allele and confirmed by DNA sequencing. Cre-mediated deletion of *CERK* exon 6 (encoding amino acids 191-238) results in a translational frame-shift with termination in exon 8. Mice carrying the KO allele were subsequently bred to C57BL/6J mice, and Cre-negative offspring were selected to establish the *CERK* -KO line. **(B)** RNA from WT and *CERK* -KO primary dermal fibroblasts was converted to cDNA and used for quantitative PCR analysis using primers specific to the mouse *CERK* gene. Samples were compared using paired t-test (n=4, **p < 0.01). **(C)** Genotypic analysis of WT, Floxed, and *CERK*-KO alleles was performed using genomic DNA, analyzed via end point multiplex PCR using the following primers: Intron 5 forward primer: 5'-CTGAGATGGCCGTTTCTCACAGAG-3' (P1), intron 6 forward primer: 5'-ATGCTCACTAGAGACCCAGTCCTC-3' (P2), and intron 6 reverse primer: 5'-TTCACCAGGCTTTGGACACAGCAC-3' (p3) and examined via gel electrophoresis. When using the cassette specific primer sets, three bands are possible, WT – 210 bp (p_2-p_3), Floxed – 250 bp (p_2-p_3 containing LoxP and BamH I sites), and *CERK*-KO – 528 bp (p_1-$p_3$without exon 6).

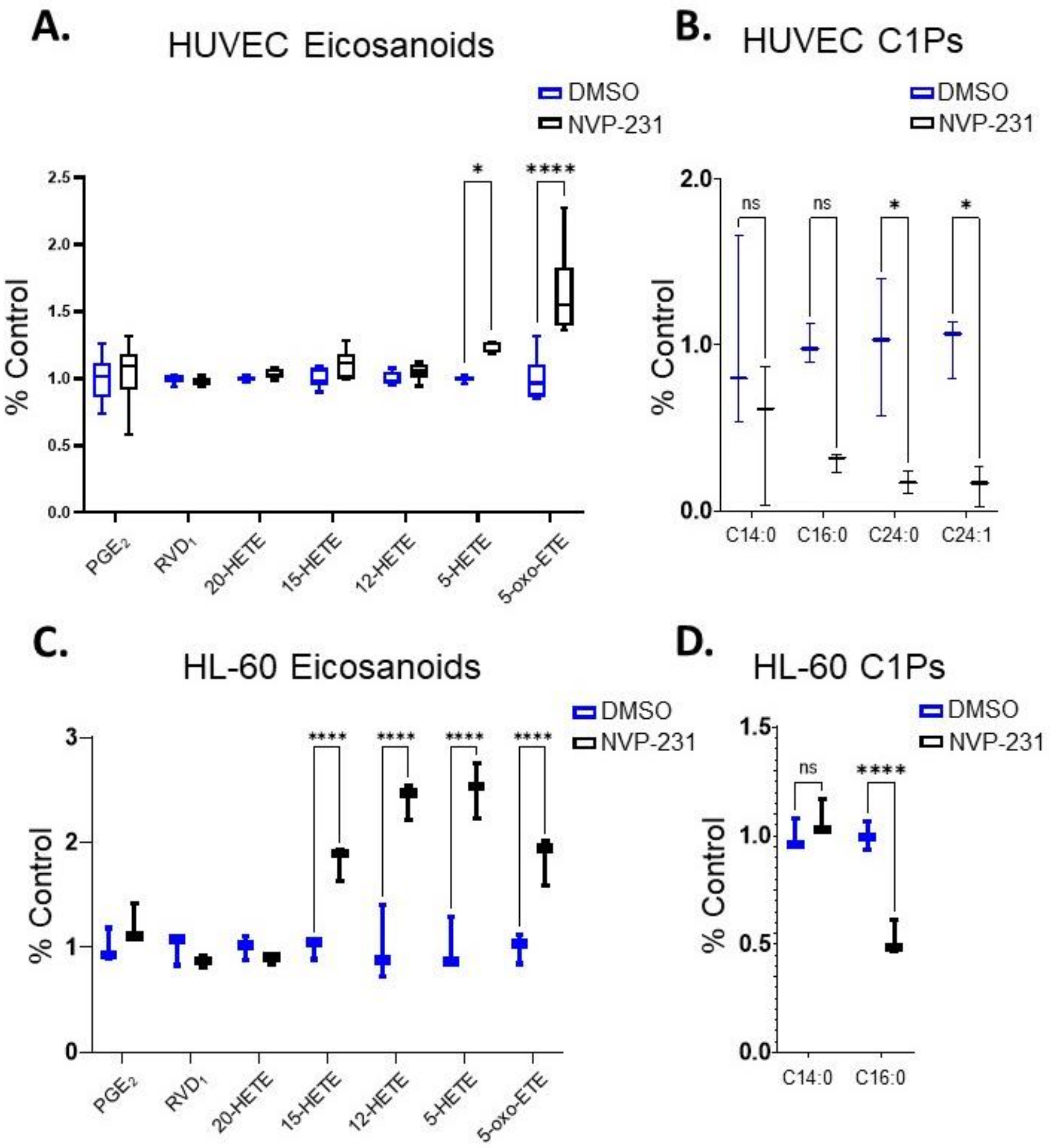

Figure S2. Eicosanoid profile of HUVEC and HL-60 cells. (A) Graphical analysis of eicosanoids (n=6) and (B) C1P species (n=3) from HUVECs pre-treated with NVP-231 (300 nM) or DMSO (0.001%) control for 24 hours. (C) Graph depicting eicosanoid (n=3) and (D) C1P species from HL-60 cells (n=3) treated with NVP-231 for 24 hours (Two-way ANOVA with Šídák's multiple comparisons test; *$p < 0.05$, ****$p < 0.0001$).

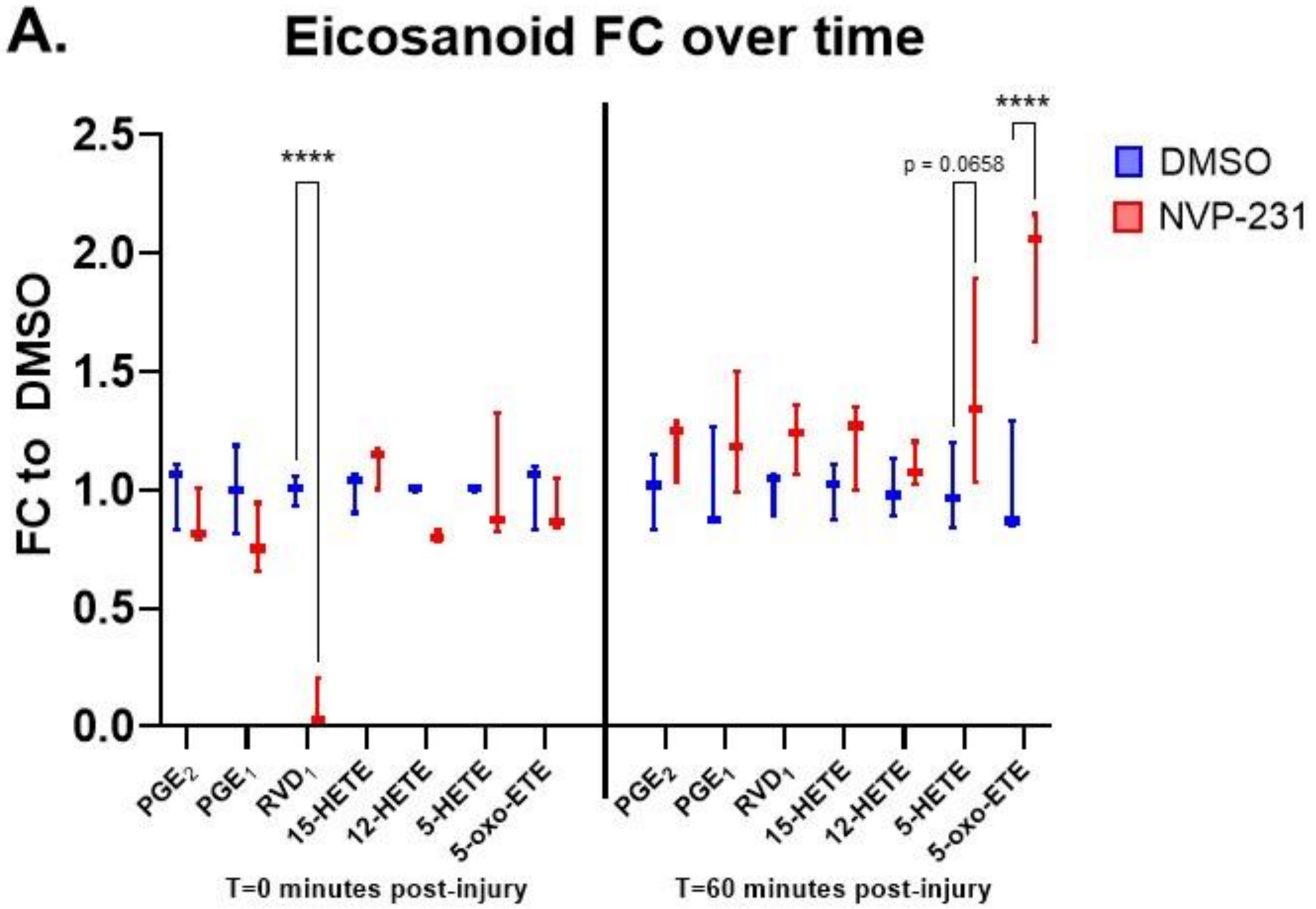
A.
Eicosanoid FC over time
FC to DMSO
2.5
2.0
1.5
1.0
0.5
0.0
****
p = 0.0658
****
DMSO
NVP-231
PGE2
PGE1
RVD1
15-HETE
12-HETE
5-HETE
5-oxo-ETE
T=0 minutes post-injury
T=60 minutes post-injury

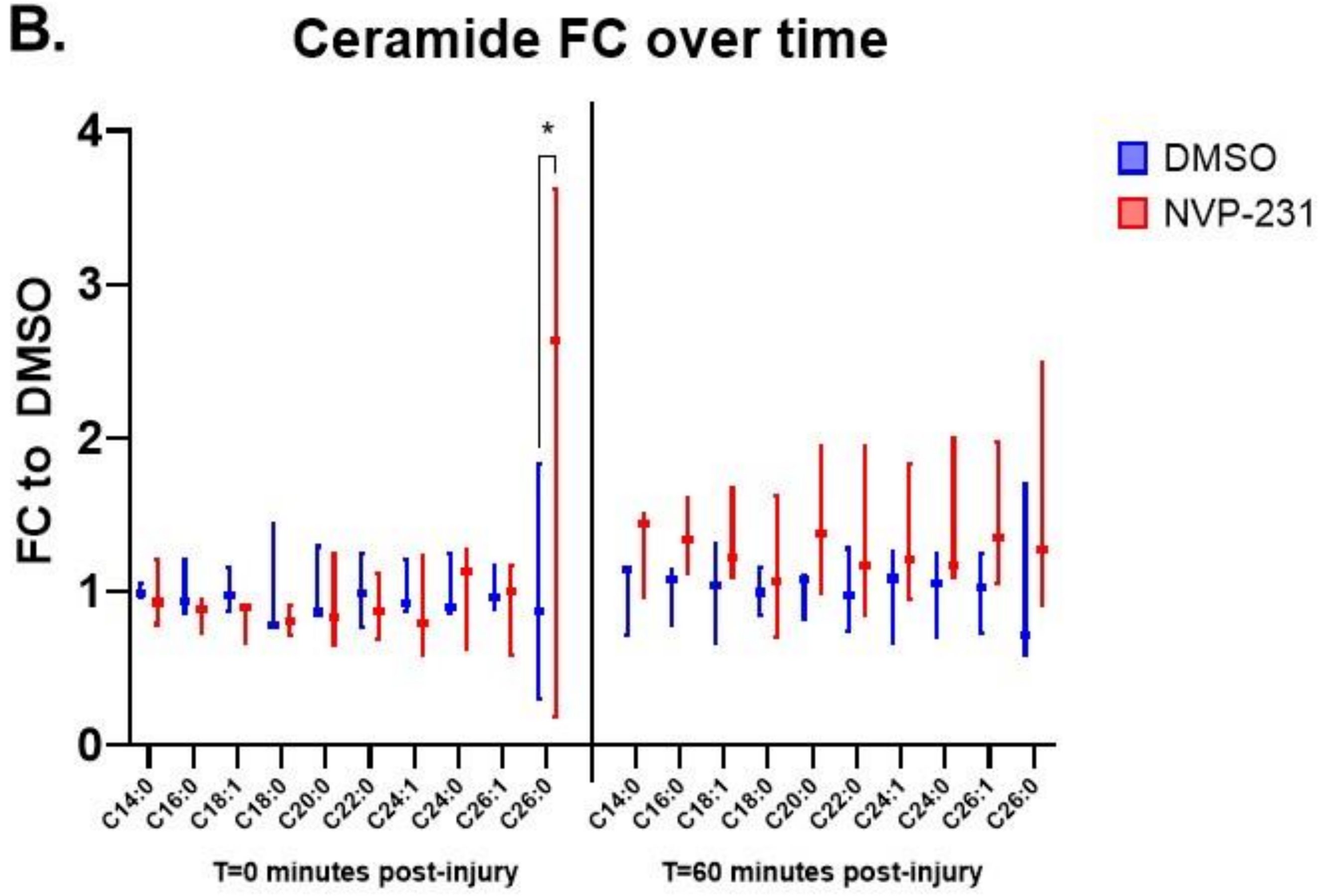
B.
Ceramide FC over time
FC to DMSO
4
3
2
1
0
*
DMSO
NVP-231
C14:0
C16:0
C18:1
C18:0
C20:0
C22:0
C24:1
C24:0
C26:1
C26:0
T=0 minutes post-injury
T=60 minutes post-injury

Figure S3. Scratch assay lipid changes over time. (A) Eicosanoid changes for wild-type pDF scratch assay at times 0- and 60-minutes post-injury (n=3). (B) Ceramide changes for wild-type pDF scratch assay at time 0- and 60-minutes post-injury (n=3). Wild-type pDFs were pre-treated with DMSO (0.01%) or NVP-231 (100 nM) for 30 minutes received mechanical trauma via asterisk pattern scratch across the plate. Cells were collected immediately (T=0) or 60-minutes (T=60) post-injury and analyzed for eicosanoid and ceramide levels via UPLC ESI-MS/MS. Values expressed as fold change to DMSO controls (*$p < 0.05$, ****$p < 0.0001$; n=3, pDFs collected from 3 different mice. Two-way ANOVA with Šídák's multiple comparisons test).
